# Smear- and Polymerase Chain Reaction (PCR)-Negative Tuberculosis: A Case Series Highlighting Diagnostic Limitations and the Role of Escalation

**DOI:** 10.7759/cureus.85085

**Published:** 2025-05-30

**Authors:** Dimitri Daraselia, Ani Toloraia, Salome Tsertsvadze, Marika Todua

**Affiliations:** 1 Internal Medicine, Tbilisi State Medical University, Tbilisi, GEO; 2 Medicine, Tbilisi State Medical University, Tbilisi, GEO; 3 Innere Medizin, Christliches Krankenhaus Quakenbrück (CKQ), Quakenbrück, DEU

**Keywords:** diagnostic delay, diagnostic escalation, pcr-negative tb, pleural tb, pulmonary tb, smear-negative tb

## Abstract

Tuberculosis (TB) remains a major global health concern, and its diagnosis can be particularly challenging when microbiological tests yield negative results. We present two diagnostically complex cases, one pulmonary and one pleural, in which tuberculosis was confirmed only after extended clinical evaluation and diagnostic escalation, resulting in delays of approximately eight weeks and two weeks, respectively. The first patient, a 31-year-old asymptomatic man with recent TB exposure, underwent bronchoalveolar lavage (BAL), with smear microscopy and polymerase chain reaction (PCR) both negative; cultures remained negative after eight weeks. He was later readmitted with hemoptysis, and repeat BAL ultimately yielded a positive culture for *Mycobacterium tuberculosis*, despite persistently negative PCR.

The second patient, a 22-year-old woman presenting with pleuritic chest pain and a large unilateral pleural effusion, had markedly elevated inflammatory markers and exudative fluid on thoracentesis. Initial smear, PCR, and bronchoscopy were inconclusive. A definitive diagnosis of pleural TB was established only after thoracoscopic biopsy revealed necrotizing granulomatous inflammation. In both cases, early identification of bacterial co-infections contributed to diagnostic delay. These cases highlight the limitations of conventional diagnostics in both pulmonary and pleural TB, particularly in smear- and polymerase chain reaction (PCR)-negative presentations. They underscore the importance of clinical vigilance and timely escalation to tissue-based diagnostics, including repeat bronchoscopy and thoracoscopy, when initial evaluations are non-diagnostic. A structured, multimodal approach is essential to minimize diagnostic delays and ensure early initiation of appropriate therapy.

## Introduction

Tuberculosis (TB) remains a major global health challenge, consistently ranking among the leading infectious causes of morbidity and mortality worldwide. While pulmonary TB is the most common clinical form, extrapulmonary manifestations also contribute significantly to the overall disease burden [1,2]. Diagnosing TB can be particularly challenging when the clinical presentation is atypical, symptoms are non-specific, or the bacterial load is low, as in paucibacillary disease. In such cases, standard diagnostic tools-including acid-fast bacilli (AFB) smear microscopy and nucleic acid amplification tests (NAATs)-frequently yield negative results, leading to missed diagnoses or substantial delays in treatment initiation [3,4].

These diagnostic delays are clinically significant, as they have been associated with poorer outcomes, including treatment failure, loss to follow-up, and increased mortality risk [5]. Further complicating the diagnostic picture, co-infection with common respiratory pathogens can obscure the underlying etiology and mislead the clinical assessment, particularly in patients without traditional TB risk factors [6-8]. Radiological and laboratory findings in TB are often inconclusive, and definitive diagnosis typically depends on microbiological culture or histopathological confirmation, both of which may require invasive procedures and extended turnaround times [9-11].

In this case series, we present two adult patients with diagnostically complex presentations who were ultimately diagnosed with tuberculosis after inconclusive initial investigations. These cases are particularly illustrative as they involve immunocompetent patients with co-infections, a scenario that further complicates diagnosis and can lead to significant delays. These cases underscore the limitations of smear and PCR testing in both pulmonary and extrapulmonary TB and highlight the importance of maintaining clinical vigilance. They illustrate the need for timely diagnostic escalation when early results are non-diagnostic, regardless of the site of disease, and reinforce the value of a persistent, multimodal approach in real-world practice. It is important to note that both cases occurred in a high-resource setting with ready access to advanced diagnostics, including thoracoscopy and prolonged mycobacterial culture, which may not be representative of challenges faced in low-resource contexts.

## Case presentation

We present two adult patients with different clinical backgrounds who were ultimately diagnosed with pleural tuberculosis following inconclusive initial investigations. In both cases, early microbiological testing failed to identify *Mycobacterium tuberculosis*, and definitive diagnosis was delayed until histopathological confirmation or culture conversion. These cases underscore the diagnostic limitations of smear and PCR-based methods and highlight the value of persistent, multimodal evaluation in suspected tuberculosis.

Case 1

A 31-year-old male from Pakistan was referred from a refugee reception facility due to potential exposure to a confirmed tuberculosis case. He was asymptomatic on presentation, reported no prior medical history or complaints, and denied any respiratory symptoms at the time of screening. Physical examination was unremarkable, and baseline laboratory values, including complete blood count, liver and renal function, and inflammatory markers, were within normal limits (Table 1).

**Table 1:** Initial laboratory results on admission Reference ranges reflect the standards used at Christliches Krankenhaus Quakenbrück (CKQ), Quakenbrück, Germany. WBC – white blood cell count, CRP – C-reactive protein, Hb – hemoglobin, Hct – hematocrit, PTI – prothrombin index, pCO₂ – partial pressure of carbon dioxide, pO₂ – partial pressure of oxygen, SpO₂ – oxygen saturation.

Parameter	Result	Reference Range	Interpretation
WBC (Leukocytes)	14.4 × 10⁹/L	4.0 – 11.0 × 10⁹/L	↑ Leukocytosis
Platelets	463 × 10⁹/L	150 – 400 × 10⁹/L	↑ Thrombocytosis
C-Reactive Protein (CRP)	122.2 mg/L	< 5 mg/L	↑ Markedly elevated (suggests inflammation)
Hemoglobin (Hb)	13.4 g/dL	13.0 – 17.0 g/dL (men)	Normal
Hematocrit (Hct)	39.5%	40 – 50% (men)	Slightly ↓ but likely clinically insignificant
Prothrombin Index (PTI)	74%	80 – 120%	↓ Mildly reduced (may suggest coagulopathy)
Sodium (Na⁺)	142 mmol/L	135 – 145 mmol/L	Normal
Potassium (K⁺)	4.1 mmol/L	3.5 – 5.1 mmol/L	Normal
Serum Lactate	10.7 mg/dL	4.5 – 19.8 mg/dL	Normal
Arterial pH	7.41	7.35 – 7.45	Normal
pCO₂	33.5 mmHg	35 – 45 mmHg	Mildly ↓ (respiratory alkalosis or compensation)
pO₂	64.5 mmHg	80 – 100 mmHg	↓ Mild hypoxemia
Oxygen Saturation (SpO₂)	97%	95 – 100%	Normal

**Figure 1:**
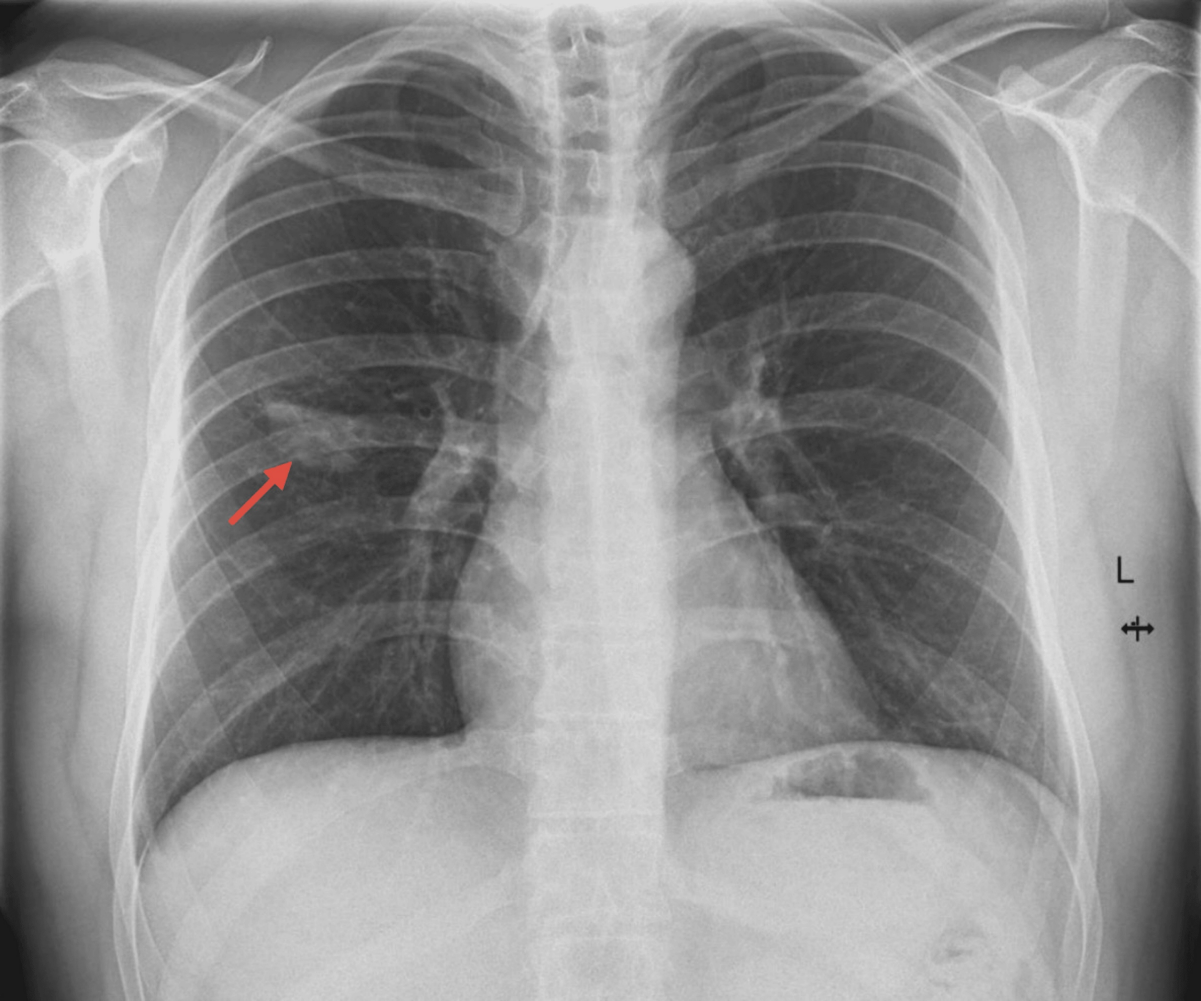
Chest X-ray (PA view) The image shows an infiltrate located in the right middle lobe (labeled by the red arrow). PA - posteroanterior.

**Figure 2:**
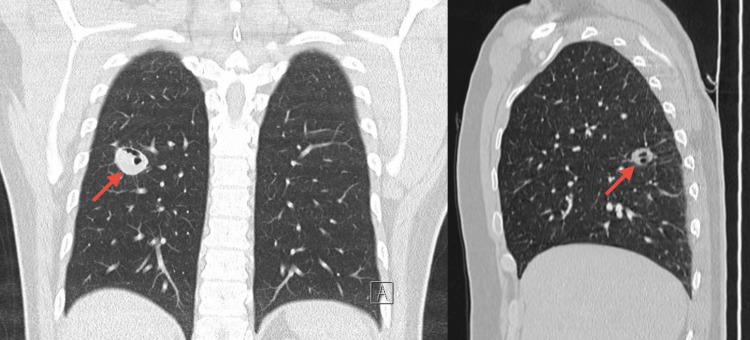
Chest CT scan (coronal and sagittal views) with contrast The scan was obtained with intravenous contrast, and it revealed a cavitary lesion in the right middle lobe (red arrows).

Initial routine X-ray revealed an infiltrate located in the right middle lobe (Figure 1). A thoracic CT scan confirmed the presence of a cavitary structure in the same region (Figure 2).

**Figure 3:**
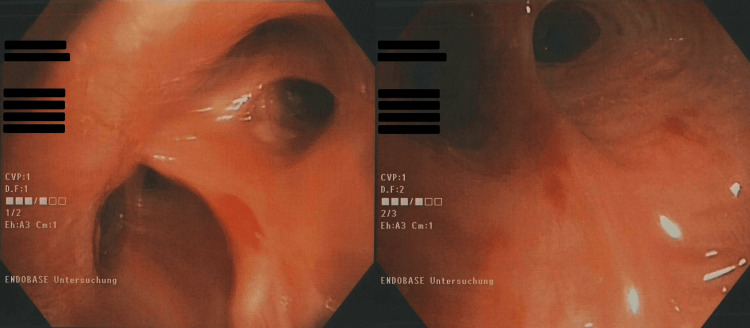
Bronchoscopic examination of upper and lower airways Flexible bronchoscopy revealed normal anatomy and mucosal appearance. The hypopharynx and larynx showed no signs of irritation, and the trachea appeared normal. The main carina was sharp and moderately ridged, with unremarkable left and right bronchial trees. No direct or indirect signs of tumor infiltration were observed down to the subcarinal level. The endobronchial system was assessed as normal. Image obtained at Christliches Krankenhaus Quakenbrück. Used with institutional permission.

A decision was made to perform bronchoscopy with bronchoalveolar lavage (BAL) (Figure 3). Direct smear microscopy was negative for acid-fast bacilli, and PCR for *Mycobacterium tuberculosis* complex DNA was also negative. BAL fluid analysis showed the presence of *Staphylococcus aureus* at a concentration of 2 × 10⁴ CFU/mL. Antibiotic therapy was initiated with clindamycin and cefotaxime. The culture for *Mycobacterium tuberculosis* was still pending.

At the eight-week follow-up, BAL cultures remained negative for *Mycobacterium tuberculosis*. Given the negative smear, PCR, and initial culture results, coupled with the identification of *Staphylococcus aureus*, the patient was discharged with a presumptive diagnosis of bacterial pneumonia and continued outpatient antibiotic therapy. Approximately two months later, the patient was readmitted with hemoptysis and underwent a repeat bronchoscopy. No follow-up imaging was performed before readmission, as the initial decision was based on the negative microbiological findings and clinical improvement from his initial symptoms. At that time, the culture for *Mycobacterium tuberculosis* complex turned positive, although smear microscopy remained negative. Despite ongoing antibiotic therapy, the patient's condition persisted, and BAL cultures subsequently identified methicillin-resistant *Staphylococcus aureus* (MRSA) at a high concentration of 5 × 10⁵ CFU/mL. Notably, PCR testing for *Mycobacterium tuberculosis* remained negative even on the BAL sample that later yielded a positive culture. The final diagnosis of pulmonary tuberculosis was made based on positive culture growth. 

Case 2

A 22-year-old previously healthy, non-smoking woman presented with a four-week history of worsening general condition, exertional dyspnea, and left-sided pleuritic chest pain. She was actively weaning from breastfeeding at the time of symptom onset and reported no prior medical illnesses or known tuberculosis exposure. On clinical examination, the patient appeared undernourished but was alert and fully oriented. Pulmonary auscultation revealed diminished vesicular breath sounds and dullness to percussion at the left base. The remainder of the physical examination was unremarkable. Vital signs were stable, with a blood pressure of 102/69 mmHg, heart rate of 100 beats per minute (bpm), respiratory rate of 24 breaths per minute, and temperature of 36.8°C.

Laboratory studies demonstrated leukocytosis (14.4 × 10⁹/L), thrombocytosis (463 × 10⁹/L), and markedly elevated C-reactive protein (122.2 mg/L). Hemoglobin and hematocrit were within normal limits at 13.4 g/dL and 39.5%, respectively. The prothrombin index was mildly reduced at 74%. Electrolytes, including sodium (142 mmol/L) and potassium (4.1 mmol/L), and serum lactate (10.7 mg/dL) were within normal ranges. Arterial blood gas analysis on room air showed a pH of 7.41, pCO₂ of 33.5 mmHg, and pO₂ of 64.5 mmHg, with an oxygen saturation of 97%.

**Figure 4:**
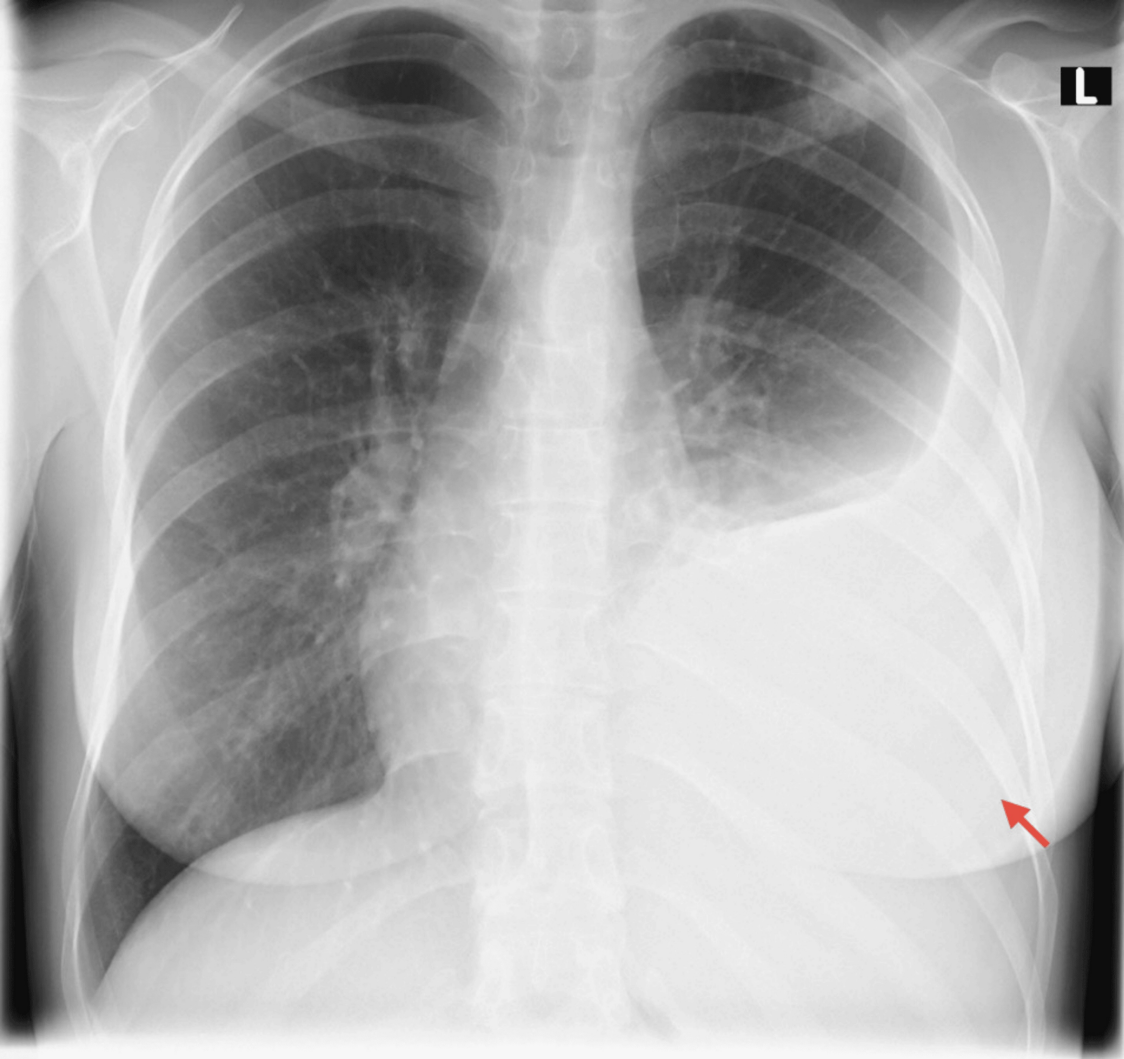
Chest X-ray at the presentation The image is demonstrating a large left-sided pleural effusion with blunting of the costophrenic angle (red arrow). This finding guided the subsequent thoracentesis and highlighted the need for diagnostic escalation given the patient's clinical presentation.

**Figure 5:**
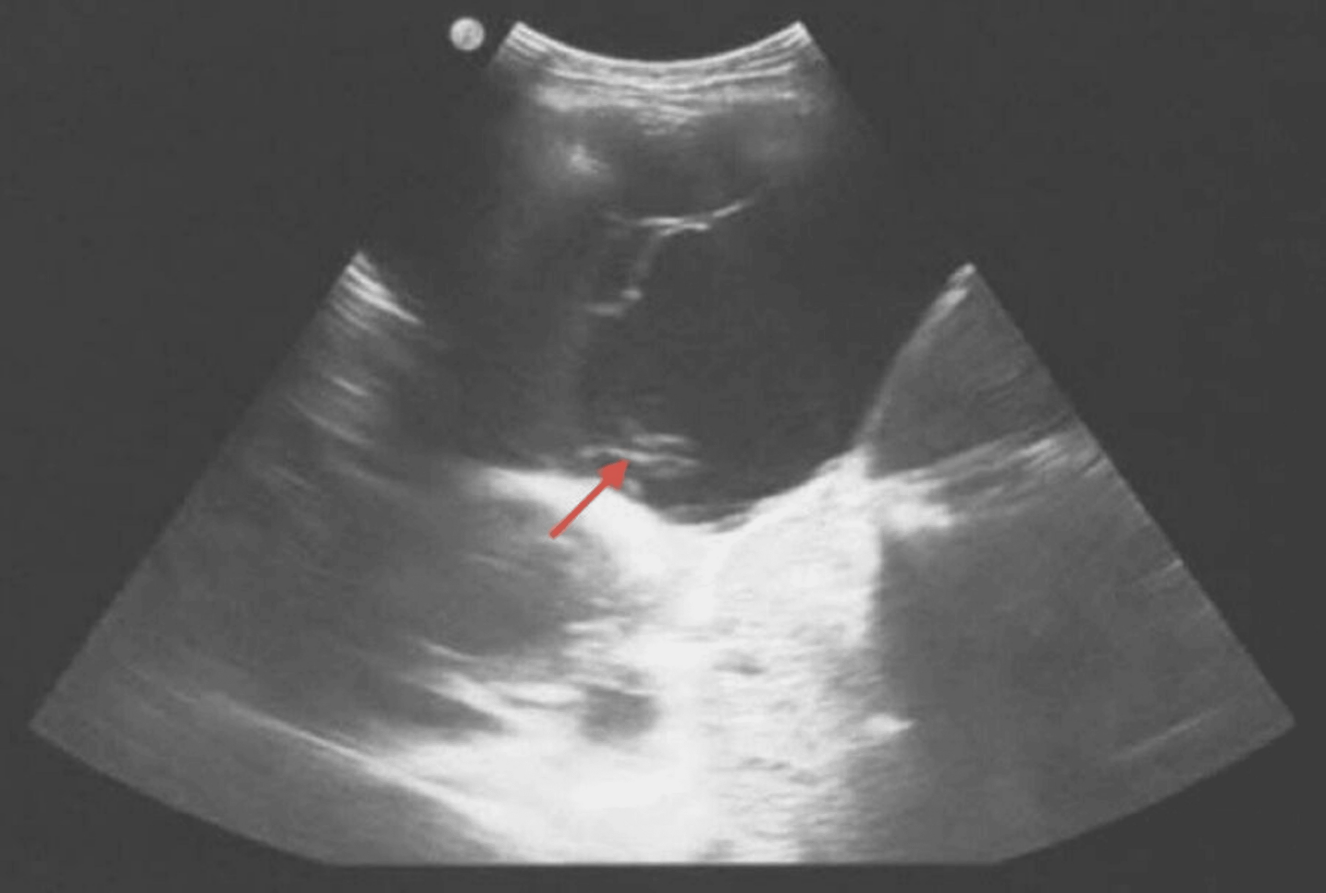
Thoracic ultrasound demonstrating significant left-sided pleural effusion Point-of-care ultrasound of the left lateral thorax reveals a markedly rising anechoic pleural effusion (echo-poor area) consistent with a large fluid collection. Ultrasound-guided thoracentesis yielded 2000 mL of yellow, slightly cloudy effusion. Approximately 1000 mL of residual fluid remained post-procedure, with noted pleural traction at the lower lobe. Image obtained at Christliches Krankenhaus Quakenbrück. Used with institutional permission.

Chest X-ray at presentation demonstrated a large left-sided pleural effusion with blunting of the costophrenic angle (Figure 4). Thoracic ultrasound identified a large anechoic pleural effusion on the left. Thoracentesis yielded 2000 mL of yellow, slightly turbid fluid, and an estimated 1000 mL of residual fluid remained post-procedure (Figure 5). Pleural fluid analysis revealed a pH of 7.73, elevated protein concentrations (4.8 and 4.1 g/dL), increased lactate dehydrogenase (LDH) levels (308 and 200 U/L), and glucose levels of 68 and 79 mg/dL across two samples. Leukocyte counts were elevated (2228 and 2500 cells/μL), and the fluid had a specific gravity of 1.015. Urea and lipase levels in the fluid were 28 mg/dL and 17 U/L, respectively (Table 2). Notably, Adenosine Deaminase (ADA) testing was not performed on the pleural fluid because it is not a routinely available test at our institution for this clinical scenario. Similarly, GeneXpert® testing on pleural fluid was not initially performed as it is primarily validated for sputum samples, and its sensitivity in pleural fluid is lower, and initial bronchoscopy findings were negative for TB in this context. Despite the relatively normal glucose and pH values, which might suggest a non-tuberculous effusion, the persistent large pleural effusion, elevated inflammatory markers, exudative nature of the fluid, and a high index of suspicion for TB in an endemic region, led to the clinical decision to proceed directly to thoracoscopy for definitive tissue diagnosis.

**Table 2:** Key abnormal laboratory and pleural fluid findings – Case 2 CRP – C-reactive protein; LDH – lactate dehydrogenase; WBC – white blood cell count; TB – tuberculosis. Reference ranges reflect the standards used at Christliches Krankenhaus Quakenbrück (CKQ), Quakenbrück, Germany. *Both the pleural fluid pH (7.73) and glucose levels (68 and 79 mg/dL) are atypically elevated for tuberculous pleuritis. These findings may reflect early disease, technical factors such as sample handling, or the paucibacillary nature of the effusion.

Parameter	Value	Reference Range	Interpretation
WBC	14.4 × 10⁹/L	4–11 × 10⁹/L	Elevated (leukocytosis)
Platelets	463 × 10⁹/L	150–400 × 10⁹/L	Elevated (thrombocytosis)
CRP	122.2 mg/L	<5 mg/L	Markedly elevated
Prothrombin Index	74%	80–120%	Mildly reduced
Protein (Pleural Fluid)	4.8 / 4.1 g/dL	0.0–3.0 g/dL	Elevated (exudative)
LDH (Pleural Fluid)	308 / 200 U/L	0–100 U/L	Elevated
Leukocytes (Pleural Fluid)	2228 / 2500 /μL	<1000 /μL	Elevated
Glucose (Pleural Fluid)	68 / 79 mg/dL*	0–50 mg/dL	Elevated (not typical for TB)
pH (Pleural Fluid)	7.73*	~7.60	Alkaline

Flexible bronchoscopy revealed normal upper airway mucosa and mild mucosal inflammation in the right bronchial tree. The left upper lobe bronchus appeared narrowed and slit-like, with partial external compression of other left-sided bronchial ostia. Cytological analysis of bronchial lavage returned as Papanicolaou Class II and showed a mixed inflammatory profile. Microbiological cultures grew *Haemophilus influenzae* and *Staphylococcus aureus*, both antibiotic-sensitive. Smear microscopy and PCR for *Mycobacterium tuberculosis* remained negative.

Due to ongoing diagnostic uncertainty and persistent pleural effusion, the patient underwent medical thoracoscopy. Intraoperatively, multiple raised, papular lesions were identified on the pleural surface. Frozen section analysis excluded malignancy. Histopathological examination of pleural biopsies revealed necrotizing epithelioid granulomas, consistent with tuberculous pleuritis.

## Discussion

Tuberculosis (TB), particularly in smear-negative and extrapulmonary forms, continues to pose a diagnostic challenge when clinical presentation is nonspecific and microbiological results are inconclusive. The two cases presented here, both initially smear- and PCR-negative, reflect this difficulty. One was eventually diagnosed following delayed culture positivity; the other required thoracoscopic biopsy for histological confirmation. These pathways reflect well-documented shortcomings of standard diagnostic tests. In a 2023 study, Wong et al. reported diagnostic yields of only 1.7% and 3.3% for AFB smears of pleural fluid and pleural biopsy, respectively, while TB-PCR had positivity rates of 14.4% in fluid and 33.3% in biopsy tissue. In contrast, cultures of pleural fluid and pleural biopsy tissue achieved yields of 53% and 69.4%, respectively [3].

These findings support the established view that histopathology, particularly thoracoscopic pleural biopsy, remains the most reliable method when non-invasive tests fail. Studies have consistently shown that thoracoscopy yields diagnostic rates exceeding 90% in pleural TB, even in cases where microbiology is negative. Histological features such as necrotizing granulomas or Langerhans-type giant cells provide decisive diagnostic confirmation in such contexts. In our second case, thoracoscopy enabled diagnosis after pleural fluid analysis, and bronchoscopy failed to detect *Mycobacterium tuberculosis*. This reflects similar outcomes reported by Haralsingh et al., McNally et al., and Boggs et al., all of whom advocate for early biopsy when pleural TB is suspected but non-confirmatory [2,9-11].

Numerous studies have warned against overreliance on smear-based diagnostic algorithms, particularly in smear-negative presentations. Swai et al. found that only 38.1% of smear-negative pulmonary TB cases were correctly identified using national diagnostic algorithms, while nearly half of true TB cases went undiagnosed [12]. Likewise, Fawibe et al. emphasized that smear-negative TB is a distinct clinical entity and not a diagnostic wastebasket; underdiagnosis and overtreatment were both common when smear was used without corroboration from culture or histology [13]. Our second case fits precisely into this pattern: all non-invasive modalities failed, and a biopsy was needed to reach a conclusive diagnosis.

A further challenge in TB diagnosis is the presence of co-infections, which often mislead clinicians toward alternative diagnoses. Pappu et al. described a case of polymicrobial pneumonia involving Klebsiella pneumoniae, *Staphylococcus aureus*, and TB, where initial focus on the bacterial pathogens delayed recognition of tuberculosis [8]. Similarly, both of our patients had positive bacterial cultures early in their hospital courses, *Staphylococcus aureus* and *Haemophilus influenzae* in one case, MRSA in the other, resulting in initial treatment with broad-spectrum antibiotics. These findings obscured the underlying TB and delayed targeted investigation (Table 3). 

**Table 3:** Common causes of diagnostic delay in smear-negative or pleural TB and recommended actions This table summarizes major contributors to the delayed diagnosis of TB, drawn from the literature. PCR – polymerase chain reaction; TB – tuberculosis.

Category	Cause of Delay	Supporting References	Recommended Action/Escalation
Patient-Related	Co-infection masking TB	6-8	Consider co-infection resolution, but maintain high TB suspicion; re-evaluate for TB if symptoms persist after initial antibiotic course.
	Atypical radiology	2,11,17	Perform advanced imaging (CT) and consider early invasive sampling if suspicious findings persist.
	Misclassified as non-TB illness	7,14,18	Re-evaluate diagnosis with a low threshold for TB testing in endemic areas or with risk factors, especially if not responding to empiric therapy.
System-Related	Negative PCR/smear (low sensitivity)	1,3,13	Do not exclude TB based on single negative smear/PCR; pursue culture and histology, especially if clinical suspicion is high.
	Delay in biopsy/escalation	9,11,15	Timely escalation to invasive procedures (e.g., thoracoscopy, biopsy) when non-invasive tests are inconclusive and suspicion remains.

Smear-negative TB, though often underappreciated, accounts for a substantial portion of cases and is frequently associated with diagnostic delays. McNally et al. reported that microbiological confirmation is obtained in just 15.5% of pleural TB cases, requiring clinicians to rely on histology or clinical judgment in the majority of cases [2]. This aligns with the findings of Boggs et al., who described a case with negative smear, PCR, and even adenosine deaminase (ADA), yet was confirmed as TB through pleural biopsy and culture [10]. Our case series reinforces the point that neither a normal ADA nor a negative PCR excludes TB in patients with exudative pleural effusions and systemic symptoms.

Delays in TB diagnosis are not merely academic, they have serious clinical consequences. Kraef et al. found that diagnostic delays exceeding one month were associated with increased mortality among patients with HIV [14]. Although our patients were immunocompetent, both experienced delayed diagnoses, one of whom had required readmission for hemoptysis before the underlying etiology was confirmed (Table 4). This echoes the findings of Nakao et al., who described TB cases initially misdiagnosed as aspiration pneumonia and confirmed only after clinical deterioration and delayed culture positivity [11]. Similarly, Asres et al. demonstrated that delays beyond 30 days from symptom onset significantly increased the risk of treatment failure, death, or loss to follow-up, with an adjusted relative risk of 1.92 for unsuccessful outcomes [5].

**Table 4:** Comparative diagnostic timeline for Case 1 and Case 2 This chart illustrates the key events and approximate duration from initial presentation to definitive tuberculosis diagnosis for both patients. It highlights periods of diagnostic uncertainty and the impact of co-infections. Abbreviations: BAL – bronchoalveolar lavage; PCR – polymerase chain reaction; MRSA – methicillin-resistant Staphylococcus aureus; TB – tuberculosis.

Timeline Event	Case 1	Case 2	Time to Definitive TB Diagnosis (Days from Initial Presentation)
Presentation & suspicion of TB	After exposure in refugee center	After 4 weeks of symptoms	0
Initial labs/smear/PCR	BAL smear and PCR negative	Thoracentesis and PCR negative	Approx. 3-7 days
Co-infection identified	S. aureus	S. aureus, H. influenzae	Approx. 3-7 days
Initial treatment	Cefotaxime + clindamycin	Empiric antibiotics	(Ongoing)
Second bronchoscopy or escalation	Repeat BAL	Thoracoscopy	Case 1: Approx. 60 days (from initial BAL)
Definitive TB diagnosis	Positive culture	Histology positive	Case 1: Approx. 60 days
			Case 2: Approx. 14 days (from initial thoracentesis)

The value of repeat testing and diagnostic escalation is a central theme across the literature. In a case described by Dave et al., false-positive resistance testing led to inappropriate therapy until culture and specialist input resolved the diagnosis [15]. While our cases did not involve resistance, they underscore the importance of re-evaluating the diagnosis when initial tests are negative and clinical suspicion remains high. Our approach, employing bronchoscopy, repeated culture, thoracoscopy, and histopathology, mirrors the stepwise strategy supported by authors such as Dheda, Hara, and Pinto, all of whom highlight the frequency with which TB mimics community-acquired pneumonia and resists first-line diagnostics [7,16-18].

Quantitatively, smear-negative TB remains a significant burden. In a Nepalese cross-sectional study, Khadka et al. found that 23.6% of patients with smear-negative presumptive TB were confirmed via GeneXpert®, and 40.4% of those had pleural effusions, underscoring the radiographic and clinical overlap between TB and other infections [4]. Our second case falls squarely within this category, with pleural effusion and elevated inflammatory markers prompting a non-TB workup until thoracoscopy clarified the diagnosis.

Altogether, our case series aligns with consistent findings across the literature, emphasizing that pleural tuberculosis is frequently smear- and PCR-negative, that co-infections may obscure the underlying diagnosis, and that histopathological evaluation, particularly via thoracoscopy, is often the most definitive diagnostic tool. In patients presenting with persistent exudative effusions, inconclusive microbiological testing, or epidemiologic risk factors such as residence in high-burden settings, clinicians should maintain a high index of suspicion and escalate to tissue-based diagnostics when warranted. A stepwise, multimodal approach remains essential to reduce diagnostic delay and ensure timely treatment.

Study limitations

As a two-case series from a single high-resource center, our findings may not generalize to all patient populations or settings, especially those with limited access to advanced diagnostics like thoracoscopy or prolonged cultures. We also acknowledge the absence of specialized tests like pleural fluid ADA in Case 2. While highlighting diagnostic challenges, the observed delays are specific to these presentations and may not reflect all smear-negative TB cases.

## Conclusions

These two cases underscore the limitations of conventional microbiological testing in smear- and PCR-negative tuberculosis and highlight the diagnostic uncertainty that arises in the setting of co-infection and nonspecific clinical findings. In patients with unexplained exudative pleural effusions, tuberculosis should remain high on the differential, even in the absence of microbiologic confirmation. Early thoracoscopic biopsy offers the most definitive path to diagnosis and should not be delayed when clinical suspicion persists. Ultimately, a structured, multimodal escalation strategy, anchored in clinical vigilance, is essential for the timely and accurate diagnosis of pleural TB.
